# Topology Optimisation of Wideband Coaxial-to-Waveguide Transitions

**DOI:** 10.1038/srep45110

**Published:** 2017-03-23

**Authors:** Emadeldeen Hassan, Daniel Noreland, Eddie Wadbro, Martin Berggren

**Affiliations:** 1 Department of Computing Science, Umeå University, Umeå, SE-901 87, Sweden; 2 Department of electronics and electrical communications, Menoufia University, Menouf, 32952, Egypt

## Abstract

To maximize the matching between a coaxial cable and rectangular waveguides, we present a computational topology optimisation approach that decides for each point in a given domain whether to hold a good conductor or a good dielectric. The conductivity is determined by a gradient-based optimisation method that relies on finite-difference time-domain solutions to the 3D Maxwell’s equations. Unlike previously reported results in the literature for this kind of problems, our design algorithm can efficiently handle tens of thousands of design variables that can allow novel conceptual waveguide designs. We demonstrate the effectiveness of the approach by presenting optimised transitions with reflection coefficients lower than −15 dB over more than a 60% bandwidth, both for right-angle and end-launcher configurations. The performance of the proposed transitions is cross-verified with a commercial software, and one design case is validated experimentally.

Coaxial-to-waveguide transitions have been designed using a variety of techniques. By trial and error, Wheeler^1^ placed metallic blocks close to the transition plane inside the waveguide to obtain a wideband operation. Later, theoretical analysis, based mainly on transmission line theory, has been used to model and design various transitions^2^,^3^. The transitions were typically modelled by assembling sections of coaxial cables, coaxial waveguides, ridge waveguides, or rectangular waveguides. Currently, it is possible to design transitions using full-wave numerical solutions to Maxwell’s equations^4^. However, most of the proposed transitions still depend essentially on the concept of heuristically cascading various transmission line sections^5^,^6^,^7^,^8^, with only few parameters to optimise. The complexity involved in assembling various sections, especially when 3D structures are used, can complicate mass producibility. Simeoni *et al*.^9^ proposed using patch antennas as compact transitions suitable for mass production. However, the use of canonical shapes (circular patches) limits the operational bandwidth to at most 25%, even when two stacked patches are used.

In this work, we take a completely different approach to the design of coaxial-to-waveguide transitions. We use the method of *topology optimisation*, which quite recently has been developed to a stage that makes it useful for this kind of design challenge. The revolutionary aspect of this approach is that the conceptual operational principle of the device will not, as in previous studies, be decided in advance by trial and error or by cascading elementary sections but will be a *result* of the optimisation. As we will see, this approach will generate wideband transitions with much simpler layouts compared to existing wideband devices. The optimised transitions are easy to fabricate and provide excellent matching.

“Topology optimisation” is the name most commonly used for a technique to determine, with the help of a gradient-based numerical optimisation algorithm, the arrangement of materials in a given domain such that a prescribed objective is achieved. The most common approach to topology optimisation is the *material distribution* (also called *density-based*) approach. Here, the idea is to use a very large number of design variables (at least >10^3^; the current record in structural mechanics is ∼10^9^!) to create a pixel (2D) or voxel (3D) “image” of the design. If the image resolution is fine enough—and this is a crucial requirement—the number of potential designs is almost limitless and the approach can be used for *conceptual design*; the operational principle of the device will emerge from the design process. (Compare with photography, where a very high pixel count is needed for high quality!). The possibility to operate with 10^3^–10^9^ design variables within a reasonable computational time (here we use about 200 design cycles) relies on a high utilisation of simulation data through the so-called *adjoint-field* approach. When evaluating the performance of a device, we are usually interested in performance measures such as the reflection coefficient or the input impedance at some ports. Nevertheless, in simulations, we actually obtain much more information, namely the field values at each point of the computational grid. The adjoint-field approach utilises these internally computed field values together with the field values of an additional system of equations, the adjoint equation, to compute sensitivity information for all design variables. (The adjoint equation is here also the Maxwell equations, but with a different forcing that depend on the choice of objective function.) Using this approach, it is possible, with only one extra solution of a system of equations (the adjoint equations) per design cycle, to compute sensitivity information for all design variables. That is, information on how a change of *each pixel individually* improves a performance measure is obtained in *one* sweep (forward plus adjoint equations), independent of the number of design variables. This is in stark contrast to the dominating metaheuristic optimisation methods, such as genetic algorithms and swarm optimisation, routinely used for electromagnetic problems. Such algorithms solely rely on samples of the performance measure for each design configuration and do not utilise distributed sensitivity information, which makes these methods too computationally costly for large-scale optimisation problems^10^.

Topology optimisation, which was initially developed to design load-carrying elastic structures^11^,^12^, has had a significant impact on the field of mechanical design, particularly in the car and aerospace industries, and is by now available in several commercial codes for structural analysis. The approach has been successfully extended to other disciplines, including acoustics^13^,^14^ and optics^15^. For design problem within electromagnetics, the development has been much slower. The layout design of dielectric materials using topology optimisation can be carried out with a similar approach as for mechanics problems^16^,^17^. However, metallic devices are different, due to *the ohmic barrier problem*: a small region of material is lossless in the limits of zero or infinite conductivity, whereas substantial ohmic losses appear for intermediate values. A straightforward implementation of standard material distribution methods for topology optimisation along the line developed for mechanics problems will therefore not work; the ohmic barrier will effectively prevent the optimisation algorithm to change from conductor to air or vice versa. However, strong algorithmic developments during the last few years makes it now possible to invoke topology optimisation also for the design of two- as well as three-dimensional metallic devices in electromagnetics^18^,^19^,^20^,^21^,^22^,^23^,^24^,^25^,^26^,^27^. In particular, the current group of authors have developed an approach based on imposed ohmic losses, through a so-called design filter^28^,^29^. Design filters are routinely applied in material distribution based topology optimisation to regularise the problem, ensure mesh-independence, and avoid numerical instabilities. In contrast, in our approach, the main purpose of the filter is to impose ohmic losses, which relaxes the strong self-penalisation of the optimisation problem, primarily at the beginning of the optimisation process. During the optimisation process, the influence of the filter is successively reduced^23^,^24^,^25^,^26^. A Danish group pursues a similar approach for frequency-domain problems^18^,^19^,^27^, whereas we concentrate on wide-band applications using time domain methods.

Here we show that the newly developed topology approach for metallic electromagnetic devices can be tuned to the design of coaxial-to-waveguide transitions. The idea is to place a piece of circuit board vertically in the middle of a rectangular waveguide to which a 50 Ohm coaxial cable is attached, either in a side-launcher or right-angle configuration. The topology optimisation algorithm will then work out the shape of the etched copper on one side of the circuit board in order to maximise the coupling of signals between the waveguide and the coaxial cable. We emphasise that in this approach, the operational principle, which will turn out to be a radiating lump, possibly together with a back reflector for the side-launcher, or a tapered structure for the end-launcher is not prescribed. The current approach produces simple-shaped transitions with wideband performance that classical design methods indeed can match, but with much more complexity in their design^5^,^6^.

## Problem setup

**Figure 1:**
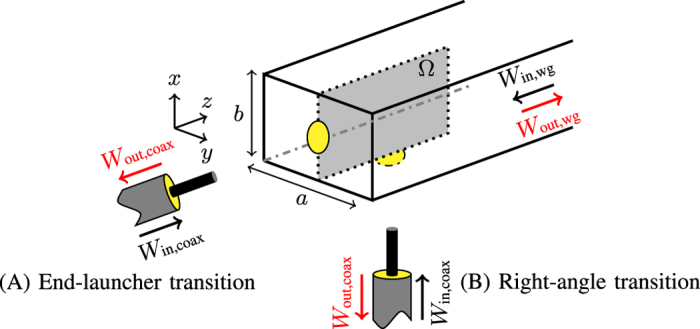
Optimising the conductivity over the design domain Ω (the grey region enclosed by the dotted line) to match the 50 Ohm coaxial cable (**A** or **B**) to a standard rectangular waveguide with cross section *a* × *b* and aligned to the *z* axis.

An *a* × *b* rectangular-waveguide aligned with the *z* axis is terminated with a conducting wall at *z* = 0 and extends to infinity in the positive *z* direction, see Fig. 1. A 50 Ohm coaxial cable is connected through the conducting wall located at *z* = 0 (cable A) or *x* = 0 (cable B). The transition formed with cable A is denoted end-launcher transition and with cable B right-angle transition. The coaxial cable probe is connected to a design domain Ω, where a conductivity distribution σ_Ω_ is to be determined such that the coaxial cable and the waveguide are matched over a specific frequency band. The domain Ω is selected to have height *b*, depth *a*, aligned to the *xz* plane, and positioned at the plane *y* = *a*/2. Moreover, the domain Ω will be backed by a low-loss dielectric substrate to hold the conductivity distribution σ_Ω_.

## Problem formulation and numerical solution

Inside the waveguide, the electric field, ***E***, and the magnetic field, ***H***, are governed by the 3D Maxwell’s equations,


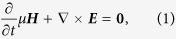



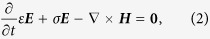


where *μ, ε*, and *σ* are the permeability, permittivity, and conductivity of the medium. Inside the coaxial cable, under the assumption that only the TEM mode is supported, the potential difference, *V*, and the current, *I*, satisfy the 1D transport equation^23^,


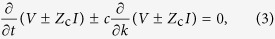


where *Z*_c_ and *c* are the characteristic impedance and the phase velocity inside the *k*-directed coaxial cable. The terms *V* − *Z*_c_*I* and *V* + *Z*_c_*I* represent signals traveling inside the coaxial cable in the negative and positive *k*-direction, respectively.

We assume that both the coaxial cable and the waveguide extend to infinity (matched). Moreover, we assume that the only signal source is an incoming energy, *W*_in,wg_, associated with the TE_10_ mode, propagating inside the waveguide towards the negative *z* axis; see Fig. 1. The incoming energy through the coaxial cable, *W*_in,coax_, is assumed to be zero. Under the above assumptions, we can write the following energy balance for the cable–waveguide system,


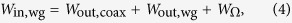


where the right side is the total outgoing energy that consists of the energy existing through the coaxial cable *W*_out,coax_, the reflected energy in the waveguide *W*_out,wg_, and the ohmic loss 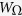 inside the domain Ω. Inspecting expression (4), we note that a natural design objective is to maximize the signal coupled to the coaxial cable (cable A or cable B), *W*_out,coax_, which implicitly implies the minimization of the remaining two terms *W*_out,wg_ and 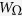. Therefore, given the incoming energy, *W*_in,wg_, we formulate the conceptual optimisation problem


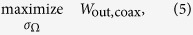


subject to the set of governing equations and boundary conditions.

We numerically solve equations (1), (2), and (3) by the FDTD method^30^. Let ***σ*** be a vector that holds the conductivity components at each Yee edge inside the domain Ω. The goal is to find the ***σ*** that maximizes the outgoing energy through the coaxial cable, which we accomplish through a gradient-based optimisation method. Let ***p*** be a vector of the same dimension as ****σ**** storing the design variables that are actually updated by the optimisation algorithm; it holds that 0 ≤ *p*_*i*_ ≤ 1 for each component of ***p***. The design variables should interpolate between the conductivity values representing a good dielectric (*p*_*i*_ = 0) and a good conductor (*p*_*i*_ = 1). However, there is a vast variation in the conductivity value between a good dielectric and a good conductor. For example, the free space has *σ* = 0 S/m and the copper has *σ* = 5.8 × 10^7^ S/m. The average of these values still represents a good conductor, so the use of a linear interpolation between these values would make the algorithm overly sensitive for small changes of almost vanishing design variables. We therefore use the following exponential interpolation scheme:


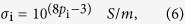


which gives *σ*_min_ = 10^−3^ S/m and *σ*_max_ = 10^5^ S/m for *p*_*i*_ = 0 and *p*_*i*_ = 1, respectively. Numerical experiments show a low sensitivity of the objective function to variations in *σ* outside the range [*σ*_min_, *σ*_max_], and the conductivity value for *p*_*i*_ = 1/2 now indeed represents a lossy material that is neither a good dielectric nor a good conductor.

Formally, our optimisation problem reads.


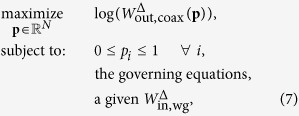


where 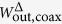 is the outgoing energy through the coaxial cable computed by the FDTD method, and *N* is the number of Yee edges inside the design domain Ω. The spectral density of the incoming energy, 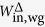, will implicitly determine the frequencies for which the structure will be optimised. To address the wideband objective function in optimisation problem (7), we use a time-domain *sinc* signal to impose the incoming energy, 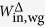, inside the waveguide. A *sinc* signal with infinite duration has a flat energy spectral density over a specific bounded frequency interval^31^. To realise a reasonable simulation time, we truncate the *sinc* signal after 8 lobes. The truncated *sinc* signal is modulated to the center of the frequency band of interest and its bandwidth is set to cover this frequency band. The objective function gradient is computed using the solution to an adjoint-field problem, which is also a FDTD discretization of Maxwell’s equations, but is fed with a time reversed version of the observed signal at the coaxial cable^32^. For any number of design variables, the gradient of the objective function can be computed with only two FDTD simulations, one to the original-field problem and one to the adjoint-field problem.

Optimisation problem (7) is strongly self-penalised towards the lossless design cases. More precisely, solving problem (7) by gradient-based optimisation methods leads, after only a few iterations, to designs consisting mainly of a good conductor (*σ*_max_) or a good dielectric (*σ*_min_). The reason for the strong self-penalisation can be explained by energy balance (4) as follows. To maximize the outgoing energy, *W*_out,coax_, for a given incoming energy, *W*_in,wg_, the energy losses, 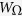, inside the design domain should by minimized. The intermediate conductivities contribute to higher energy losses than the extreme conductivities, as pointed out in the introduction. Thus, any gradient-based optimisation method will attempt to minimize the energy losses, 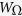, by moving the edge conductivities towards the lossless cases (*σ*_min_ and *σ*_max_). Unfortunately, the resulting optimised designs often consist of scattered metallic parts and exhibit bad performance^24^.

To relax the strong self-penalisation, we use a filtering approach that imposes some intermediate conductivities inside the design domain during the initial phase of the optimisation. To do so, we replace *p*_i_ by *q*_i_ in expression (6), where the vector ***q*** = *K**p***, in which the filter matrix *K* is a discrete approximation of an integral operator with support over a disc with radius *R*. The filter replaces each component in the vector ***p*** by a weighted average of the neighbouring components, where the weights vary linearly from a maximum value at the center of the disc to zero at the perimeter. To avoid lossy final designs, we solve optimisation problem (7) through a series of subproblems, where after the solution of subproblem *n*, the filter radius is reduced by setting *R*_n+1_ = 0.8 *R*_n_. We start with a filter radius *R*_1_ = 40Δ, where Δ denotes the FDTD spatial discretization step. Each subproblem is iteratively solved until a stopping criterion for optimisation, based on the first-order necessary condition, is satisfied. To update the design variables, we use the globally convergent method of moving asymptotes (GCMMA)^33^.

## Results and Discussions

We investigate the design of transitions between a 50 Ohm coaxial cable (probe diameter 1.26 mm, outer shield diameter 4.44 mm) and and two standard waveguides; the WR90 (*a* = 22.86 mm and *b* = 10.16 mm) and the WR430 waveguides (*a* = 109.22 mm and *b* = 54.61 mm). The first cutoff frequency of the WR90 waveguide is *f*_10_ = 6.56 GHz and the second is *f*_20_ = 13.12 GHz, while the WR430 waveguide has *f*_10_ = 1.37 GHz and *f*_20_ = 2.75 GHz. The frequency band of interest in both cases is the band between the first and the second cutoff frequencies, where only the TE_10_ mode can propagate (below, we refer to this bandwidth as the *bandwidth objective*). These frequency bands correspond to a relative bandwidth of 66.7% for the WR90 waveguide and 67.0% for the WR430 waveguide. In our numerical experiments, we use uniform FDTD grids. For the WR90 waveguide we use a spatial step size Δ = 0.127 mm, and for the WR430 waveguide Δ = 0.607 mm. In both cases, a 16 cell perfectly matched layer (PML) is used to terminate the waveguide and 15 cells of free space separate the end of the design domain and the beginning of the PML. We use an in-house FDTD code implemented to run on graphics processing units (GPU) using the parallel computing platform CUDA (https://developer.nvidia.com/what-cuda). One solution to Maxwell’s equations takes around 5 minutes and around 4 GB of memory is required for computing the objective function gradient. The waveguide walls and the coaxial probe are assigned a conductivity value of *σ* = 5.8 × 10^7^ S/m.

### Right-angle transitions

**Figure 2:**
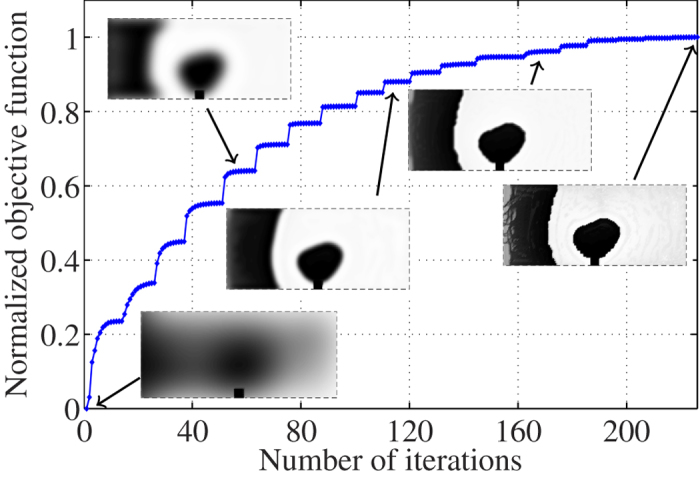
The progress of the objective function together with some snapshots that illustrate the development of the design (black colour: good conductor, white colour: good dielectric).

We start by designing a right-angle transition between the 50 Ohm coaxial cable and the WR90 waveguide. We use a design domain Ω with area 22.86 × 10.16 mm^2^, see Fig. 1, and with the coaxial feed connected at the middle of the bottom side of Ω. The design domain is backed by a low-loss RT/Duroid 5880 LZ substrate (relative permittivity 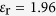, thickness = 1.27 mm, 35 *μ*m copper-clad, and tan*δ* = 0.002 at 10 GHz). The domain Ω is discretized into 180 × 80 Yee cell faces and is expected to have a mesh-dependent effective thickness of 0.2Δ = 25 *μ*m^34^. We fix an area of 10 × 10 Yee cell faces close to the coaxial probe as a conductor to provide a good contact between the coaxial cable and the domain Ω. The optimisation problem has 28,410 design variables associated with the interior Yee edges in Ω. The design process starts with a uniform conductivity *σ* = 1500 S/m. Figure 2 shows the progress of the objective function during the optimisation process together with some snapshots that demonstrate the development of the design. The black colour indicates a good conductor while the white colour indicates a good dielectric. The operation of the design filter, which is necessary to combat the ohmic barrier problem discussed in the introduction, is evident in Fig. 2. A design blurring filter enforces large amounts of losses in the beginning of the iterations to prevent large regions to be locked into a pure dielectric or metallic state^24^. The radius of the blurring is periodically reduced, which can be seen as the increasing steps in the objective function values. At each such step, the filter radius decreases. The optimisation algorithm can then increase the sharpness of the design in order to decrease the ohmic losses and improve the objective function value.

The algorithm converged after 226 iterations to the design given in the last snapshot in Fig. 2. (Note that at each iteration, the FDTD code is called, on average, 3 times for computing the objective function, the gradient, and finding suitable updates). The black colour indicates a good conductor and the white colour indicates a good dielectric. We note that the final design has a grey region behind the reflector part, near the *z* = 0 plane. Numerical investigations indicate a low sensitivity of the objective function to design variables located at that region. In addition, the scattering parameters of the transition do not change by mapping these grey values either towards a good conductor or a good dielectric. Therefore, in a post-processing step and to obtain a binary design, we map conductivity values below and above *σ* = 1 S/m to 0 S/m and 5.8 × 10^7^ S/m, respectively.

**Figure 3:**
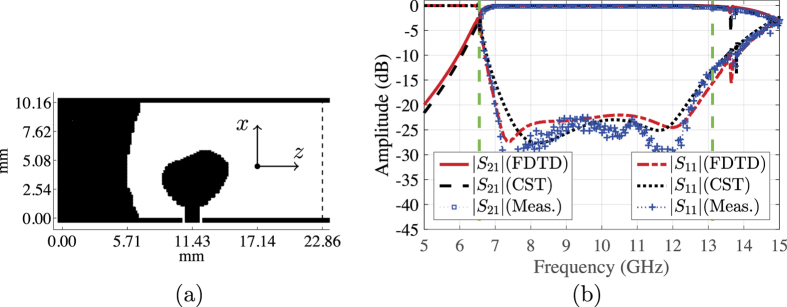
(**a**) The optimised conductivity distribution over a 22.86 × 10.16 mm^2^ design domain (right-angle transition). (**b**) The measured and simulated scattering parameters of the transition.

Figure 3a shows the final conductivity distribution over the design domain (enclosed by the boundaries of the waveguide and the dashed line). The design consists of a conductive area connected to the inner probe of the coaxial cable and a reflector part. The reflector part has one parabola-like boundary and is connected to the walls of the waveguide at the three remaining boundaries. We point out that there is a supporting dielectric substrate that holds the two conductive parts. The optimised design is fabricated and a complete transition is assembled, as discussed below in the last section. The performance of the optimised design is evaluated experimentally and through simulations. We use the commercial CST Microwave Studio package (https://www.cst.com/) to cross-verify our FDTD computations. Figure 3b shows the scattering parameters of the transition. The dashed vertical lines mark the frequency band between the first and the second cutoff frequencies, which is the frequency band of interest. There is good match between the experimental and the simulations results. The transition has |*S*_11_| lower than −15 dB and |*S*_21_| greater than −0.3 dB over the frequency band 6.85–12.89 GHz, which corresponds to a relative bandwidth of 61.2% (recall that the bandwidth objective is 66.7%).

**Figure 4:**
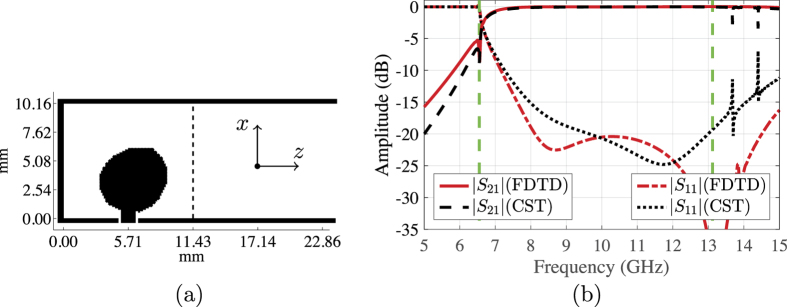
(**a**) The optimised conductivity distribution over a 11.43 × 10.16 mm^2^ design domain (right-angle transition). (**b**) The simulated scattering parameters of the transition.

Aiming for compactness, we observe that part of the design domain is used by the algorithm to build the reflector part. Therefore, we decide to cut in half the area of the previous design domain, down to 11.43 × 10.16 mm^2^ discretised into 90 × 80 Yee cell faces, which yields 14,100 design edges (excluding the edges on the waveguide walls and the fixed area close to the probe). We remark that also in this case there is a backing RT/Duroid 5880 LZ substrate with the same size as the design domain. Figure 4a shows the final design obtained by the algorithm after 220 iterations, with the design consisting only of a conductive part connected to the probe of the coaxial cable. Figure 4b shows that the optimised transition has a reflection coefficient below −15 dB and a corresponding coupling coefficient above −0.3 dB starting at 7.6 GHz and extend beyond the dominate mode frequencies. Within the dominant mode frequencies, the achieved relative bandwidth is 53.3% (recall that the bandwidth objective is 66.7%). Compared to the previous case, the separation between the probe and the shorting wall of the waveguide is decreased by half, which explains the decrease in the transition matching near the lower cutoff frequency.

**Figure 5:**
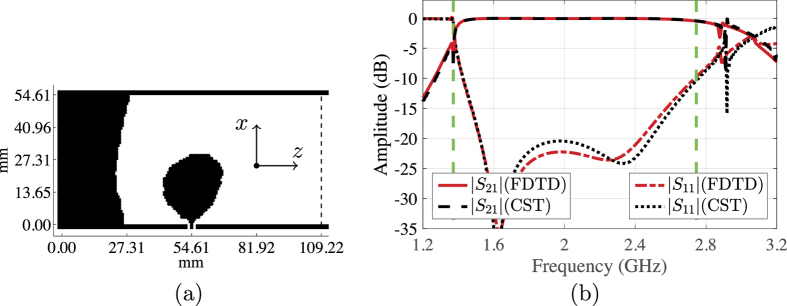
(**a**) The optimised conductivity distribution over a 109.22 × 54.61 mm^2^ design domain inside a WR430 waveguide (right-angle transition). (**b**) The simulated scattering parameters of the transition.

The fixed dimensions of the coaxial cable and the substrate thickness make it difficult to scale the transitions introduced for the WR90 waveguide to be used with another waveguide operating in a different frequency band. Therefore, we decided to design a right-angle transition between the 50 Ohm coaxial cable, with the same dimensions as before, and a WR430 waveguide. We use a design domain Ω with area 109.22 × 54.61 mm^2^ (see Fig. 1), which is discretised into 180 × 90 Yee cells. To hold the design, the domain Ω is backed with the same RT/Duroid 5880 LZ substrate as before. We fix a contact area of 2 × 10 Yee cell faces to be a good conductor, inside the domain Ω, at the probe position. The optimisation problem has 32,170 design variables. The optimisation algorithm converged in 218 iterations to the design given in Fig. 5a, where the design domain is enclosed by the waveguide boundaries and dashed line. The structure of the optimised design is similar to the transition for the WR90 waveguide, and consists of an active part connected to the inner probe of the coaxial cable and a parabola-like reflector part. Figure 5b shows the scattering parameters of the optimised transition. The transitions has a reflection coefficient below −15 dB and a corresponding coupling coefficient above −0.3 dB over the frequency band 1.47–2.56 GHz, which corresponds to a relative bandwidth of 54.1% (recall that the bandwidth objective is 67.0%).

### End-launcher transitions

**Figure 6:**
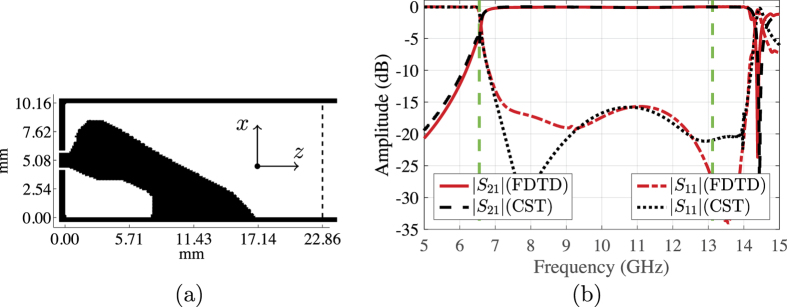
(**a**) The optimised conductivity for the WR90 end-launcher transition. (**b**) The scattering parameters of the optimised transition.

In this section, we investigate the design of end-launcher transitions, see Fig. 1. Typically, this type of transition is well suited for applications such as compact phased array antennas, where the radiating elements could be waveguide sections. We use similar settings as the right-angle transition except that the coaxial cable is connected to the domain Ω at the centre of the shorting wall at *z* = 0. First, we present a design for an end-launcher transition between the 50 Ohm coaxial cable and the WR90 waveguide. Figure 6a shows the final design obtained by the optimisation algorithm after 249 iterations. The design is connected to the probe at one side and the other side is short circuited with the bottom wall of the waveguide. The optimised transition has the upper side tapered, a design feature commonly used to achieve wideband impedance transformers^5^. In addition, we may interpret the conductivity-free region at the lower side as a wideband matching stub. Figure 6b shows that the transition has |*S*_11_| lower than −15 dB and |*S*_21_| greater than −0.3 dB starting at 7.1 GHz and extend beyond the dominant mode frequencies. Within the dominant mode frequencies, the achieved relative bandwidth is 59.6% (recall that the bandwidth objective is 66.7%).

**Figure 7:**
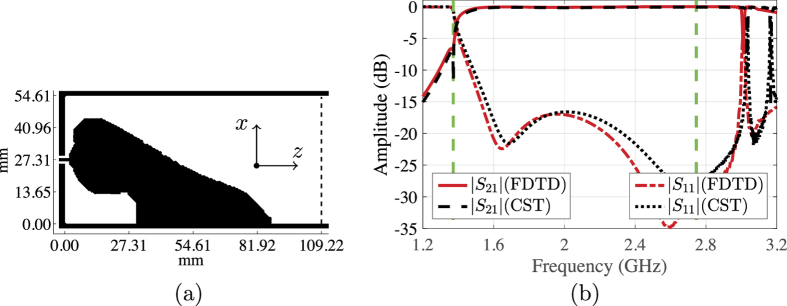
(**a**) The optimised conductivity for the WR430 end-launcher transition. (**b**) The scattering parameters of the optimised transition.

Finally, we present a design for an end-launcher transition between the 50 Ohm coaxial cable and the WR430 waveguide. We use the same setting as for the WR430 right-angle transition presented in the previous section, except that the coaxial cable is connected at the centre of the shorting wall at the *z* = 0 plane. Figure 7a shows the optimised conductivity distribution obtained by the optimisation algorithm after 231 iterations. As shown in Fig. 7b, the proposed transition has a reflection coefficient lower than −15 dB and a corresponding coupling coefficient above −0.3 dB starting at 1.53 GHz and extend beyond the dominant mode frequencies. Within the dominant mode frequencies, the achieved relative bandwidth is 50.4% (recall that the bandwidth objective is 67.0%).

## Experiments

**Figure 8:**
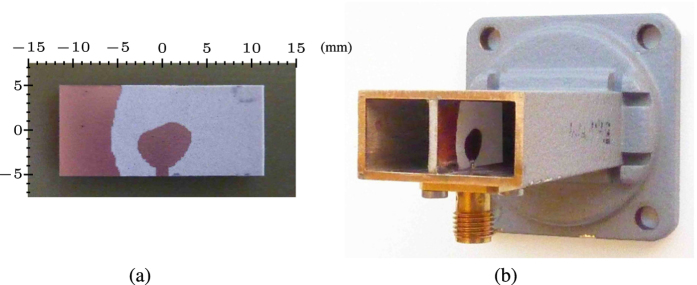
(**a**) The fabricated design. (**b**) the right-angle transition with backing plate removed for visibility.

A prototype for the right-angle transition was produced from a 56 mm long section cut from a WR90 flanged straight waveguide. The interior substrate patch was fabricated using a photoengraving procedure providing an accuracy of around 25 *μ*m. The patch (Fig. 8a) was cut out and soldered inside the waveguide using spacers, later removed, for precise alignment. The machining and mounting precision was likewise around 25 *μ*m. A standard SMA connector was used to couple the transition to a coaxial cable. To connect the active part of the antenna with the SMA connector probe without introducing an impedance mismatch, a hole of the same diameter as the jacket of the connector was drilled in the side of the waveguide. An annular Teflon liner was inserted into the hole to provide a 50 Ohm continuation for the SMA probe between the connector flange and the antenna. Figure 8b shows the finished set up. The S-parameters were measured using a sliding short. A Keysight N9918A vector network analyser was connected to the transition, in turn attached to a waveguide section with an internal sliding short of the finger connector type^35^. Assuming reciprocity and a perfect short, the reflection coefficient Γ seen from the SMA connector side is related to the S-parameters through the relation 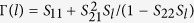, where *S*_*l*_ = −exp(−*i*2*βl*) is the reflection coefficient of the sliding short component, with the short placed at position *l*, and *β* is the frequency dependent wavenumber of the dominant TE_10_ mode. After measuring Γ for 15 values of *l* spaced 2.5 mm apart, the S-parameters were found using non-linear regression. For validation, *S*_11_ was also measured directly with the transition connected to a matched load, with very similar results.

## Conclusion

We used a topology optimisation approach to design coaxial-to-rectangle waveguide transitions. The approach allows designs to be found, from scratch, without any other geometrical assumptions than the outer dimensions. This design freedom can potentially find shapes that would otherwise be very difficult to conceive. The proposed transitions are suitable for mass production by standard microstrip technology, and require only one assembly step for installation inside the waveguides. One of the designs is successfully validated experimentally, with good agreement between simulations and experimental results.

## Additional Information

**How to cite this article:** Hassan, E. *et al*. Topology Optimisation of Wideband Coaxial-to-Waveguide Transitions. *Sci. Rep.* **7**, 45110; doi: 10.1038/srep45110 (2017).

**Publisher's note:** Springer Nature remains neutral with regard to jurisdictional claims in published maps and institutional affiliations.
